# A Native Bee, *Melissodes* *tepaneca* (Hymenoptera: Apidae), Benefits Cotton Production

**DOI:** 10.3390/insects11080487

**Published:** 2020-08-01

**Authors:** Isaac L. Esquivel, Robert N. Coulson, Michael J. Brewer

**Affiliations:** 1 Department of Entomology, Texas A&M University, College Station, TX 77843, USA; r-coulson@tamu.edu; 2 Department of Entomology, Texas A&M AgriLife Research, Corpus Christi, TX 78406, USA; mjbrewer@ag.tamu.edu

**Keywords:** *Melissodes tepaneca*, cotton yield, pollination

## Abstract

The cotton agroecosystem is one of the most intensely managed, economically and culturally important cropping systems worldwide. Native pollinators are essential in providing pollination services to a diverse array of crops, including those which have the ability to self-pollinate. Cotton, which is autogamous, can potentially benefit from insect-mediated pollination services provided by native bees within the agroecosystem. Examined through two replicated experiments over two years, we hypothesized that native bees facilitated cross-pollination, which resulted in increased lint of harvested bolls produced by flowers exposed to bees and overall lint weight yield of the plant. Cotton bolls from flowers that were caged and exposed to bees, flowers that were hand-crossed, and bolls from flowers on uncaged plants exposed to pollinators had higher pre-gin weights and post-gin weights than bolls from flowers of caged plants excluded from pollinators. When cotton plants were caged with the local native bee *Melissodes tepaneca,* seed cotton weight was 0.8 g higher on average in 2018 and 1.18 g higher on average in 2019 than when cotton plants were excluded from bees. Cotton production gains from flowers exposed to *M. tepaneca* were similar when measuring lint and seed separately. Cotton flowers exposed over two weeks around the middle of the blooming period resulted in an overall yield gain of 12% to 15% on a whole plant basis and up to 24% from bolls produced from flowers exposed directly to *M. tepaneca.* This information complements cotton-mediated conservation benefits provided to native pollinators by substantiating native bee-mediated pollination services provided to the cotton agroecosystem.

## 1. Introduction

Pollination services provided by managed and native bees (Hymenoptera: Apidae) are vital in the production of many crops. Insects, particularly bees, pollinate 66% of the world’s 1500 crop species and are directly or indirectly essential for an estimated 15–30% of crop production [1]. Although cotton is an autogamous species, meaning it has the ability to self-pollinate, it can still benefit from insect-mediated pollination. Other autogamous crops such as coffee, canola, and soybean see considerable gains when visited by bees [2]. For example, coffee showed increases in yield from 14% to 50% when surrounding pollinator-friendly vegetation was a source for pollinators in coffee [3]. Cotton has large flowers that produce large amounts of pollen and nectar that can serve as a food resource and attractant for many pollinating insects, including native bees. However, cotton pollen is rather large and too heavy to move between flowers without the assistance of an insect courier [4,5,6]. For native bee conservation, the availability of mass flowering crops such as cotton across agricultural landscapes often has a positive impact on the density of generalist, native bee pollinators [7]. 

The cotton agroecosystem is one of the most intensely managed, economically and culturally important cropping systems worldwide. China, India, Pakistan, Brazil, and the United States comprise the top five cotton producers in the world. In the U.S., more than 20 million bales of cotton were produced in 2017 worth over 7 billion USD in value. Cotton is produced in the Cotton Belt, spanning the southern region of the U.S. from Virginia to California, including Texas [8]. Out of this region, 45% of U.S. cotton is produced in Texas. Cotton was once managed with an intensive insecticide regimen to control heliothine pests (Lepidoptera: Heliothinae) and the cotton boll weevil (*Anthonomus grandis*, Boheman, Coleoptera: Curculionidae). With the development of Bt cotton to control the heliothine complex and the boll weevil eradication program, there has been a substantial reduction in insecticide usage to control cotton pests, particularly in the U.S. [9]. The availability of cotton flowers and an overall decrease in insecticide usage could be a potential benefit to local native bee pollinators. Native bees have been observed to nest in and visit cotton frequently [10], and the potential for bee conservation is high [7]. Unfortunately, the pollinator status of these native bee species is relatively unknown, as is the potential for benefit to cotton. The available literature on pollinators in U.S. cotton is roughly 30 years old, and studies were completed prior to these substantial changes in cotton management. 

Much of the work done in the U.S. was conducted in the Texas Panhandle and neighboring Arizona to identify potential pollinators for economically feasible cottonseed production where cross-pollination is critical [4]. These studies found that native pollinators in the genera *Bombus*, *Agapostemon*, *Diadasia*, *Melissodes,* and *Perdita*, along with the honeybee (*Apis mellifera* L., Hymenoptera: Apidae) were found visiting cotton flowers [11,12,13]. Despite the diversity of bees found within cotton, Moffett et al. [4] reported that only three species of *Melissodes* and *A. mellifera* were found in high enough numbers to be effective pollinators. Despite evidence that native pollinators were present in cotton, the most common insect pollinator found in hybrid cottonseed production studies from the 1970s until the 2000s was *A. mellifera* [14]. 

In a review investigating pollination benefits to cotton yield, Cunningham et al. [14] reported that all 31 studies analyzed indicated pollination benefits to cotton production by bees. Interestingly, in 23 out of the 31 studies, *A. mellifera* was the species of interest. Although *A. mellifera* is prevalent in the cotton literature, using managed bees for cotton production outside of the cottonseed production industry is uncommon. There have been studies that indicate cotton harbors a diverse community of potential pollinators, including beetles, flies, and native bees, and diversity increases with cotton bloom density and the abundance of natural habitat present [15]. The bee genus *Melissodes* appears to be common in the few studies that investigate native pollinators in cotton. For example, three species in the genus *Melissodes* were found to be relatively abundant in Pima cotton fields in Arizona [4]. In Brazil, *Melissodes nigroanea* has been found to be the most abundant species of native bee in cotton [16]. *Melissodes nigroanea* has also been recorded in studies that indicate benefits to cotton flowers left exposed to pollinators compared to caged flowers in Brazil [2]. One study suggests more native bee activity in cotton fields as honeybee captures were lower than captures of the most common native bee, *M. tepaneca* [17]. Further, relatively few honeybees have been observed within cotton fields in South Texas, despite the area being a prime habitat for feral honeybees [18].

Pollinator benefits to cotton yield have been shown in Texas and other cotton-growing regions around the world. In Texas, Cusser et al. [15] indicated that caged flowers supplemented with outcrossed pollen produced higher seed cotton weights than caged self-crossed and uncaged flowers. Further, they found that there was no difference between caged, self-crossed flowers and uncaged flowers. This suggests a need for insect-mediated pollination. The trends of increased seed cotton weights and pollinator exposure were also seen in Burkina Faso, West Africa [19], and Brazil [2], where increased seed and fiber weight was linked to pollinator presence and diversity. However, these studies did not control flower exposure to pollinators directly, and only pollinator presence correlated to yield increased. 

Producers growing cotton for fiber do not currently utilize managed pollinators (e.g., honeybees or bumblebees), nor do they commonly use conservation practices that promote the visitation of wild pollinator communities in the southern U.S. In our search of the literature, previous studies of the use of and linkage of a specific native bee pollinator for benefits to cotton yield and production are rare and predate recent cotton production practices. With the reduction of insecticide usage in modern cotton production systems, we hypothesized that native bees have the potential to benefit cotton production. Any cotton production benefits from native bee pollinators would be a welcomed reciprocal benefit of cotton-mediated conservation benefits to native bees. Here we aimed to investigate the benefits of a single native pollinator, *M. tepaneca,* found within the cotton agroecosystem, on cottonseed and fiber weight. 

## 2. Materials and Methods

### 2.1. Native Bee Pollination Benefits to Cotton Yield

Replicated field experiments were conducted in 2018 and 2019 in a cotton field managed under standard practices. Randomized complete block designs with four treatments and five replications in 2018, and 10 replications in 2019, were used to investigate the benefits of the native bee *Melissodes tepaneca* on cotton fiber and seed yield. The four treatments consisted of control flowers (unmanipulated flowers on uncaged plants), caged flowers (unmanipulated flowers caged before anthesis), hand-crossed-pollinated flowers (flowers caged before anthesis, emasculated, then hand-pollinated with pollen from nearby plants), and bee-pollinated flowers (flowers caged with *M. tepaneca*). We note that in the caged flowers not exposed to *M. tepaneca*, some remnant insects (primarily sucking bugs [9]) may have been trapped inside at the time of covering the plants. However, these insects have not been noted as cotton pollinators in the literature [3,5,11,16]. Further, although direct pollination observations were not done for *M. tepaneca* within the experimental plots, native bees and other potential pollinators were observed in the cotton field where the experiment was conducted and in cotton fields within the region [4,10,15,17]. Experiments were conducted at the Texas A&M AgriLife Research and Extension Center in Corpus Christi, TX. For both years, a series of three cotton fields were planted in sequence every two weeks in order to provide us with different timings of peak cotton bloom coinciding with the source fields of the native bees used in the studies. For each year, only one planting was used for the experiment, the field approaching peak bloom that corresponded with the peak bloom of the source fields. All plantings and crop management were done using Phytogen PHY-333-WRF (Dow Agrosciences, Indianapolis, IN) and standard agronomic procedures for the region [20]. 

For each experimental setup, groups of four cotton plants were marked and randomly assigned a treatment in each replicated block. The groups of plants were left uncaged until first bloom to control for pre-bloom insect pests according to regional practices [20] and allow for growth unconstrained by the caging material. We note that caging occurred before anthesis was observed at the field level, but a few flowers may have been present. If this occurred, it was even across all treatments, and the few bolls produced would be minor contributors to total yield. Upon first bloom, groupings of four plants were caged using large organza fabric (152 × 122 cm, 240 JoAnn’s Fabrics, Hudson, OH, USA) according to their assigned treatment. These whole plant cages were successful in not inhibiting plant growth while excluding insects in other studies involving plant bugs [21]. Plants designated as experimental controls were left uncaged throughout the experiment and were exposed to any local pollinators. 

The bee species used for the caged pollinator treatment was *M. tepaneca,* which was most common in the area of the experiment and elsewhere within Texas and Mississippi [10]. Managed *Apis mellifera* hives were in the area of the experiment, about 0.25 km from the experiment in another field, but were not observed at high levels in the experimental plots. *Melissodes tepaneca* was observed in the study area at low levels (personal observation, I.L.E.). Therefore, *M. tepaneca* used in the experiment were sourced from a large commercial farming operation (source field), approximately 25 km from the experimental plots, where they were known to occur [10]. The area was monitored using bee bowls to detect when bees were actively flying in the fields along with visual observations. Bee bowls have been shown to be the most cost-effective and easy to use method to detect native pollinators in agricultural systems [22,23]. Once bees were detected in cotton at peak bloom, actively foraging *M. tepaneca* were hand-collected within cotton fields using standard aerial nets (BioQuip, Compton, CA, USA). Bees were placed in individual plastic tubes (25-dram 38 × 84 mm, BioQuip, Compton, CA, USA) and stored in a cooler with ice. Once a total of at least 25 bees were captured, they were brought back to the laboratory near the field experiment and sorted for placement into cages. Before bees were introduced into cages, cages were removed to tag candles (flower buds that WOULD open the following morning) and white flowers (anthetic flowers) to identify flowers on the plant available for bee visitation. Five bees were placed in the cages for a period of 48 h. After 48 h, another set of flowers were tagged, and another set of bees were placed into the cages. This process was repeated for a period of two weeks to maximize the number of flowers exposed to bees aligning with an appropriate four-week period of maximum flower exposure and boll set contributing to yield. Approximately one to five candles/flowers per cage were tagged at each introduction of *M. tepaneca*. Over a period of two weeks, flowers were exposed to 25 *M. tepaneca*. Cages were checked for bee activity after their introduction. Bees were seen actively foraging on flowers multiple times throughout the duration of the experiment. 

Hand cross treatments were started the same week as bees were caged with pollinators to ensure similar boll comparisons. Hand crossing entailed tagging pre-bloom flower buds (candle stage) the afternoon before manually cross-pollinating by hand. Since cotton is hermaphroditic, tagged bolls to be hand crossed were emasculated—male reproductive parts were carefully removed—to ensure no self-fertilization took place. Tagged flower buds were cross-pollinated early next morning with pollen taken from surrounding white flowers on different cotton plants. Flowers were hand-pollinated at the same pace as the bee introductions, generally one to four flowers per cage. Cotton has indeterminate growth, and flowering over a month-long period contributes to the vast majority of yield. Therefore, our experimental bee and hand-pollination manipulations were done daily for two weeks during this period in order to reflect bee visitation at our source fields and half the period of flowering which contributes to cotton lint yield. 

### 2.2. Measurements and Analysis

Once bolls matured and plants were defoliated per standard practices in preparation for harvest, bolls from the experimental plants were hand-harvested to obtain whole plant yield weight measurements. Measurements included seed cotton weight (pre-ginned cotton containing seed and fiber), lint weight (post-ginned fiber), and seed weight (post-ginned seed). In 2018, all bolls from plants were collected, weighed (Sartorius Quintix 313-1S mg balance, Sartorius, Goettingen, GER), and analyzed as an aggregate of individual bolls in each of the four plants per treatment and replication. In total, 85 bolls from hand-crossed-pollinated flowers, 92 bolls from bee-pollinated flowers, 118 caged flowers not exposed to *M. tepaneca*, and 135 uncaged flowers were used for analysis. 

In 2019, all bolls on the plant were collected for comparison (whole plant yield reported on a per-boll basis) across treatments, similar to 2018. On a whole plant basis, 382 bolls from bee-pollinated plants, 369 bolls from hand-crossed plants, 248 bolls from caged plants, and 307 bolls from uncaged plants were used for analysis. We were also interested in only bolls specifically exposed to the possible cross pollination of some of the treatments. Here, we separated tagged bolls exposed to the experimental treatments from all bolls on the plant (yield from bolls exposed to bees, hand crossed, and same aged bolls of the other treatments). Here, 170 bolls from hand-cross-pollinated flowers, 192 from bee-pollinated flowers, 148 from caged flowers, and 184 from uncaged flowers were used for analysis of yield. Further, bolls from flowers on the same plants that were not manipulated or within the same timeframe of manipulated flowers were looked at for comparison. In total, 190 bolls from flowers not exposed to bee pollinators, 199 bolls from non-hand-crossed flowers, 100 bolls from flowers on caged plants, and 123 bolls from flowers on uncaged plants were used. The intent of separate weight analysis was to consider plant resource allocation to bolls directly exposed to *M. tepaneca* or hand-crossed versus resource allocation to unmanipulated flowers. 

Analysis of yield (seed cotton, seed and lint weight) using standard ANOVA with the function “aov” in R version 3.6.0 “Planting of a Tree” [24,25] conforming to the experimental design was conducted separately for the three measurements (seed cotton weight, lint weight, and seed weight) for the whole plant yield analysis (2018 and 2019) and the additional yield weight analysis separating yields of manipulated and unmanipulated flowers (2019 only). Although multiple measurements from the same experiment were analyzed, controlling for the family-wise error rate was of minor concern, given only three measurements were considered [25]. We used related yield measurements because of their relevance to cotton production, but they experimentally were interpreted as a related group. Means across treatments for all analyses were separated using Tukey’s HSD at the α = 0.05 level when the treatment effect was significant in the ANOVA. The two separate analyses in 2019 provided a measure of the impact of pollination on the subset of individual bolls produced from flowers exposed to the treatments (response allocation to boll production potentially influenced by the treatments) compared with yield taken from bolls produced from unmanipulated flowers.

## 3. Results

### 3.1. Pollination Benefits to Whole Plant Yield via Melissodes tepaneca

**Figure 1:**
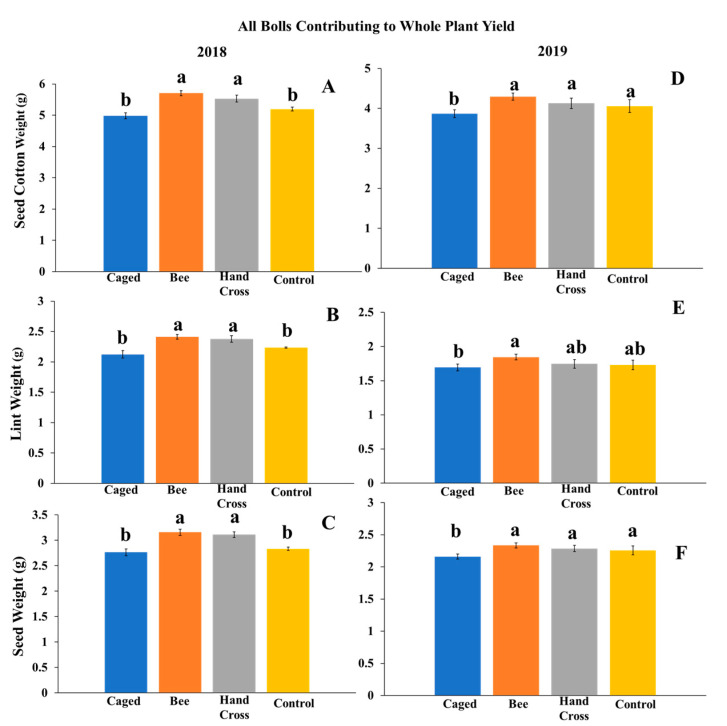
Evaluating three cotton yield measurements of whole plant yield on a per-boll basis across four treatments using all harvestable bolls on a group of 4 plants across (**A**–**C**) 2018 and (**D**–**F**) 2019. The numbers of bolls used in the analysis were as follows. In 2018: bolls from caged flowers excluded from Melissodes *tepaneca* (118), bolls from bee-pollinated flowers (n = 92), bolls from hand-crossed flowers (85), and bolls from uncaged flowers (135). “n” represents the number of bolls aggregated for analysis across the treatments. In 2019: bolls from caged flowers excluded from *M. tepaneca* (248), bolls from bee-pollinated flowers (n = 382), bolls from hand-crossed flowers (369), and bolls from uncaged flowers (307). Letters represent a significant difference at the α = 0.05 significance level using Tukey’s mean separation test.

In 2018, the analysis of variance test was significant for all three measured variables of whole plant yield recorded on a per-boll basis: seed cotton (*F* = 16.07; df = 3, 16; *p* < 0.0001), lint weight (*F* = 8.31; df = 3, 16; *p* < 0.0001) and seed weight (*F* = 17.11; df = 3, 16; *p* < 0.0001). Seed cotton weights per boll were significantly higher on average on bolls from plants with bee-pollinated flowers (5.71 g ± 0.08) and bolls from plants with hand-crossed flowers (5.53 g ± 0.1) compared to bolls from plants with uncaged flowers (5.19 g ± 0.06) and bolls from caged flowers not exposed to *M. tepaneca* (4.98 ± 0.09) (Figure 1A). This difference represented a 14.6 % increase in seed cotton weight attributable to bee exposure to flowers receptive to pollination over a two-week period. The effect was very similar when the yield was decomposed by lint and seed. Significantly higher cotton lint weights reflected a 12.3% increase in lint weight in bolls from plants with bee-pollinated flowers (2.41 g ± 0.03) and bolls from plants with hand crossed flowers (2.38 g ± 0.05) compared to bolls from plants with caged flowers not exposed to *M. tepaneca* (2.12 ± 0.04) and plants with uncaged flowers (2.23 ± 0.03) (Figure 1B). A similar increase of 14.1% of seed weight was seen in bolls from plants with bee-pollinated flowers (3.15 g ± 0.05) and bolls from plants with hand-crossed flowers (3.11 g ± 0.06) compared to plants with caged flowers not exposed to *M. tepaneca* (2.76 g ± 0.06) or uncaged flowers (2.83 g ± 0.03) (Figure 1C). 

In 2019, the analysis of variance test was significant for all three measured variables of whole plant yield recorded on a per-boll basis: seed cotton (*F* = 6.94; df = 3, 36; *p* < 0.0001), lint weight (*F* = 4.99; df = 3, 36; *p* < 0.0001), and seed weight (*F* = 5.82; df = 3, 36; *p* < 0.0001). Bolls from plants with bee-pollinated flowers (4.31 g ± 0.08), bolls from plants with hand-crossed flowers (4.12 ± 0.12), and bolls from plants with uncaged flowers (4.05 ± 0.11) all had higher seed cotton weights than bolls from plants with caged flowers excluded from *M. tepaneca* (3.54 g ± 0.09) (Figure 1D). Bolls produced from plants with bee-pollinated flowers had higher lint weight values (1.85g ± 0.04) compared to bolls from plants with caged flowers excluded from *M. tepaneca* (1.51 g ± 0.04), representing a 22% lint increase in whole plant yield attributable to bee exposure to flowers receptive to pollination over a two-week period. Bolls produced from plants with hand-crossed flowers (1.74 g ± 0.06), and bolls from plants with uncaged flowers (1.73 ± 0.07) had intermediate lint weights but did not significantly differ from the other treatments (Figure 1E). Seed weight analysis results were similar to seed cotton results: bolls produced from plants with bee-pollinated flowers (2.34 g ± 0.04), bolls produced from plants with hand-crossed flowers (2.28 g ± 0.05), and bolls from plants with uncaged flowers (2.25 g ± 0.07) all had higher seed weights than bolls from plants with caged flowers (1.99 g ± 0.04) (Figure 1F).

### 3.2. Pollination Benefits to Lint Produced by Individual Bolls from Flowers Exposed to M. tepaneca

**Figure 2:**
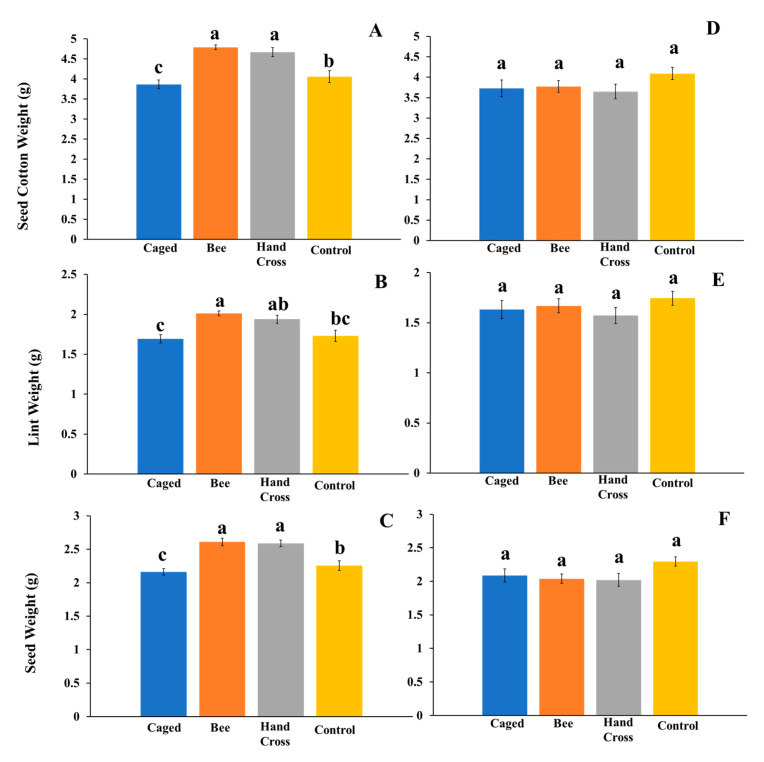
Evaluating three cotton yield measurements on (**A**–**C**) a per-boll basis using only bolls exposed to treatments in 2019 and (**D**–**F**) bolls not exposed to the treatments on the same plants. Treatments included bolls produced from flowers exposed to *M. tepaneca* and those not exposed (n = 192, n =190), and bolls from hand-crossed flowers and not-hand-crossed (n = 170, n=199), respectively. Additionally, bolls from caged flowers, in the same timeframe as treatments and those not in the same timeframe (n = 148, n= 100) respectively, and bolls from uncaged flowers in the same timeframe and those not (n = 184, n= 123), respectively. “n” represents the number of bolls aggregated for analysis across the treatments. Bolls produced from flowers exposed to treatments were tagged when placement of *M. tepaneca* occurred to ensure the comparisons at the same developmental stage of cotton growth. Letters represent a significant difference at the α = 0.05 significance level using Tukey’s mean separation test.

Data from 2019 from only bolls produced from flowers available to cross pollination and comparable bolls of the other treatments indicated significant differences across treatments for all three measured variables, recorded on a per-boll basis: seed cotton (*F* = 24.18; df = 3, 36; *p* < 0.0001), lint weight (*F* = 12.45; df = 3, 36; *p* < 0.0001), and seed weight (*F* = 11.99; df = 3, 36; *p* < 0.0001). Seed cotton weights of bolls produced from bee-pollinated flowers exhibited a 24% increase compared to bolls from caged flowers excluded from *M. tepaneca* in the same timeframe (Figure 2A). This increase is represented by the significantly different seed cotton weights of bolls produced from bee-pollinated flowers (4.790 g ± 0.06) and bolls from hand-crossed flowers (4.66 g ± 0.09) compared to bolls from caged flowers not exposed to *M. tepaneca* (3.86 g ± 0.33) and bolls from uncaged flowers (4.05 ± 0.09) (Figure 2A). Similarly, a 18.9% cotton lint weight increase was detected in bolls produced from bee-pollinated flowers compared to bolls from caged flowers not exposed to *M. tepaneca*. This is reflective of higher lint weights in bolls produced from bee-pollinated flowers (2.01 g ± 0.03), and bolls from hand-crossed flowers (1.94 ± 0.05) compared to bolls from caged flowers not exposed to *M. tepaneca* (1.69 g ± 0.15) (Figure 2B). Bolls from uncaged flowers (1.73 g ± 0.07) had intermediate lint weights from caged flowers not exposed to *M. tepaneca* and hand cross flowers (Figure 2B). In bolls produced from bee-pollinated flowers, seed weights indicated a 20.8% increase compared to those bolls from caged flowers (Figure 2C). Higher seed weights were seen in both bolls produced from bee-pollinated flowers (2.61 g ± 0.05) and those that were hand-crossed (2.58 g ± 0.04) compared to uncaged flowers (2.25 g ± 0.07). Bolls from caged flowers not exposed to *M. tepaneca* had the lowest seed weights (2.16 g ± 0.09) (Figure 2C).

Further, bolls from unmanipulated flowers were compared against each other. There were no significant differences across treatments for all three measured variables: seed cotton (F = 1.26; df = 3, 36; *p* = 0.31), lint weight (F = 0.76; df = 3, 36; *p* = 0.53), and seed weight (F = 2.05; df = 3, 36; *p* = 0.13) (Figure 2D–F). However, when both flowers exposed to treatments and flowers not exposed to treatments were added to the same model (additional “experimental treatments”), there were significant differences across all three variables: seed cotton (F = 5.51; df = 7, 72; *p* < 0.001), lint weight (F = 2.91; df = 7, 72; *p* = 0.01), and seed weight (F = 6.01; df = 7, 72; *p* < 0.001). There were significantly higher seed cotton weights of bolls from flowers exposed to *M. tepaneca* (4.79 g ± 0.06) than bolls from flowers not exposed to *M. tepaneca* (3.77 g ± 0.14) on the same plant. This represents a 27.3% difference between flowers exposed to *M. tepaneca* and those not exposed to *M. tepaneca* on the same plant. Significantly higher seed cotton weights of bolls from flowers that were hand crossed (4.66 g ± 0.09) compared to those not hand crossed (3.64 g ± 0.17) on the same plant were also seen; roughly a 28% increase/difference between bolls produced from hand-crossed and non-hand-crossed flowers.

## 4. Discussion

Our findings indicated that cotton benefited from pollination services provided by the native bee *Melissodes tepaneca*. Studies over two years reported a significant increase in seed cotton weight between 12.8% per plant and 24% per boll exposed to the native pollinators for a two-week period during peak bloom compared to plants excluded from all pollinators. The higher value of 24% was derived from data of caged bolls developed from flowers exposed to *M. tepaneca* over a period of two weeks compared to similar caged bolls positioned on plants that were excluded from pollinators. The lower value of 12.8% was derived from whole-plant yield that included all bolls harvested. This suggests that *M. tepaneca* itself plays a role in the cross-pollination of cotton flowers carrying pollen from neighboring plants within the cage or from the source field where *M. tepaneca* was collected. Although we do not know the effectiveness of these pollinators and cotton can self-pollinate, benefits from cross-pollination we detected are consistent with the literature. For example, within Texas, a study indicated increased seed cotton weights of up to 18% in flowers that were cross-pollinated with pollen from other cotton fields [15]. Although they did not use bees in their study, their hand-crossed treatment results were similar to the increase of 12.3% (2018) and 15% (2019) in whole plant yield of seed cotton weight.

Studies in other parts of the world, such as Brazil and Burkina Faso, also indicated increased yields in cotton bolls from flowers exposed to pollinators compared to pollinator-excluded flowers using different methods [2,11,26]. The Brazilian study indicated increased yields in cotton, wherein a higher diversity of pollinators was observed, including a bee in the genus *Melissodes* (*M. nigroeana*), other native bees, and *A. mellifera.* In Burkina Faso, an exclusion experiment involved caging individual flowers before bloom to allow or exclude bee visitation as an indicator of insect pollination. In flowers with observed bee visitation, higher seed cotton weights on average were seen compared to those flowers excluded from bees. They also found 27 bee species, of which *A. mellifera* and *Tetralonia fraterna* Friese (Hymenoptera: Apidae) were found to be most abundant and significantly contributed to higher cotton yields [19]. 

From a cotton producer’s perspective, interest is on the whole plant production of bolls. Previous studies mentioned above have focused on individual bolls exposed to bees or other pollinators and hand-crossed flowers. Since cotton has indeterminate growth, it has the ability to allocate resources to bolls most vital for yield production. One hypothesis is that the plants are shunting resources to cross-pollinated bolls, leaving fewer nutrients for bolls not exposed to pollinators. Based on the data from 2019 and separate analyses of manipulated and unmanipulated flowers, cross-pollination by bees—further verified by hand crossing—increased yield. The plant resources appeared sufficient to support the yield increase without affecting bolls that were not benefiting from cross-pollination by the bees. This suggests that while additional plant resources were being allocated to cross-pollinated bolls, there was not a measurable deficit in resources available to the comparable bolls that were not cross-pollinated. These are substantial findings from an agricultural viewpoint: chiefly, bees benefit cotton yield. Our additional analyses in 2019 separating yields of manipulated and unmanipulated bolls look promising. Additional experimentation on plant resource allocation and limits would be needed to explore the complexities associated with continuous flower production by cotton which affords flexibility in shifting resources during periods of stress and “opportunity” (such as availability of pollinators). Agriculturally, our results in comparing all bolls contributing to yield indicated that the cotton plant as a whole benefits from pollination by *M. tepaneca* or other pollinators that may be present locally, even during a two-week period of native bee exposure, as seen in our experiment.

It has been shown that the diversity and abundance of native pollinators in cotton agroecosystems increased when semi-natural habitats were present in both Texas and Brazil [15]. In these studies, the most abundant pollinator was in the genus *Melissodes*. In Texas, *M. tepeaneca* was found to be most abundant (species used in our study), and in Brazil, *M. nigroaenea* was most abundant [16]. A key difference in our study is that the *M. tepaneca* sourced for our experiments were taken from large scale commercial cotton fields with relatively little semi-natural habitats present compared to those monitored by Grando et al. [16]. In our large-scale source fields, *M. tepaneca* was readily collected. Therefore, this cotton benefit may be functioning both in small (less than 200 hectares) and large-scale (greater than 200 hectares) cotton fields that vary in the availability of semi-natural habitat for the bees. 

Cotton itself could potentially be beneficial for native pollinators such as those in this study and in the genus *Melissodes,* in addition to the semi-natural habitat. Further, it has been shown that mass flowering crops can enhance pollinator densities at larger scales [7]. Cotton is a mass flowering crop which has been shown to attract a broad array of potential pollinators, such as native bees, flies, and other nectar-feeding insects [27,28]. The possible reciprocal benefit of cotton providing resources to *M. tepaneca* warrants further investigation. We have shown that cotton benefits from pollination by native bees, specifically, in our case, *M. tepaneca*. We have also shown that in addition to exposure to *M. tepaneca*, seed cotton, lint, and seed weights were significantly higher in flowers that were hand crossed and those exposed to pollinators that may have been available in the uncaged treatment, compared to those excluded from pollinators using our caging technique (Figure 2A–C). This suggests that cotton can benefit from exposure from pollinators in the environment, such as *M. tepaneca*, compared to cotton devoid of pollinators. 

## 5. Conclusions

Our data, along with other studies in the region and worldwide, indicate that cotton does benefit from pollination services via native bees or honeybees. We have shown that a specific bee species that is native and is abundant in large-scale cotton fields helps increase seed cotton weight by up to 24% and lint weight by 20.8%. The data show that bee pollination of approximately half of the cotton flowers per plant triggers a yield increase significant enough to be detected at the whole-plant level. Consequently, cotton growers may benefit from strategies to promote native bee populations around their fields. These positive changes in both lint and seed production can be economically important to cotton growers. Further, it appears cotton itself provides resources for these pollinators. However, more research is necessary to investigate this potential win–win scenario for insect pollination services in terms of yield and native bee conservation.
